# The use of motion detectors to estimate net usage by householders, in relation to mosquito density in central Cote d’Ivoire: preliminary results

**DOI:** 10.1186/1756-3305-7-96

**Published:** 2014-03-06

**Authors:** Benjamin G Koudou, David Malone, Janet Hemingway

**Affiliations:** 1 Liverpool School of Tropical Medicine, Pembroke Place, Liverpool L3 5QA, UK; 2 Centre Suisse de Recherches Scientifiques en Cote d’Ivoire, 01 BP 1303, Abidjan 01, Cote d’Ivoire; 3 UFR Science de la Nature, Université Nangui Abrogoua, 02 BP 801, Abidjan 02, Cote d’Ivoire; 4 Innovative Vector Control Consortium, Liverpool School of Tropical Medicine, Pembroke Place, Liverpool L3 5QA, UK

**Keywords:** Data loggers, Net usage, Tied, Untied, Unused, Mosquitoes, Central Cote d’Ivoire

## Abstract

**Background:**

The difficulty of accurately assessing LLIN use has led us to test electronic data logging motion detectors to provide quantitative data on household LLIN usage.

**Methods:**

The main movements associated with an LLIN when appropriately used for malaria control were characterised under laboratory conditions. Data output from motion detectors attached to the LLINs associated with these specific movements were collated. In preliminary field studies in central Cote d’Ivoire, a pre-tested and validated questionnaire was used to identify the number of days householders claimed to have slept under LLINs. This information was compared to data downloaded from the motion detectors.

**Results:**

Output data recording movement on the x, y, and z axes from the data loggers was consistently associated with the specific net movements. Recall of LLIN usage reported by questionnaires after a week was overestimated by 13.6%. This increased to 22.8% after 2 weeks and 38.7% after a month compared to information from the data loggers. Rates of LLIN use were positively correlated with *An.gambiae s.s* biting density (LRT = 273.70; *P* < 0.001).

**Conclusion:**

This study showed that motion detectors can be used to provide a useful quantitative record of LLIN use. This new methodology provides a supplementary means of surveying bed net usage.

## Background

The major burden of malaria falls on the poorest quartile of the population, particularly in sub-Saharan Africa, where an estimated 627,000 people die annually from the disease [1]. African governments spend more than 1% of their gross domestic product (GDP) to combat malaria and the estimated annual direct and indirect costs attributable to malaria in sub-Saharan Africa are in excess of US$ 12 billion [2]. African Heads of State committed to a target of 80% of pregnant women and children under five being protected from malaria transmission by the use of Long Lasting Insecticides Treated Nets (LLINs) by 2010.

Sleeping under LLINs reduces malaria-related morbidity and mortality. For example in Kenya increased LLIN coverage from 6% to 67% was correlated with a 44% reduction in overall child mortality [3,4]. However, the presence of an LLIN, the status of an LLIN, and a history of sleeping under an LLIN on the night or week preceding a clinical survey, were not significantly associated with *Plasmodium* prevalence 12 months after net distribution [5,6]. Many studies speculate that pyrethroid insecticide resistance may be reducing the effectiveness of LLINs. However discrepancies between studies may be affected by rates of LLIN use, as the presence of an LLIN does not guarantee usage [5]. A recent study in Côte d’Ivoire recorded an increase in *Plasmodium* prevalence 9 months after net distribution, which could be due to LLIN failure or the inappropriate reported rate of LLIN use by their owners [6]. A study in western Kenya found that 30% of LLIN recipients did not use the nets properly [7]. Sleeping under an LLIN the night before a survey is the main indicator used by Roll Back Malaria to estimate net usage rates. This indicator is generally considered accurate and predictive of net usage. However, reports from Niger and Ethiopia indicated the rates of utilization may be overestimated. Usage may also change over time, for example, in a survey conducted during the dry season with low mosquito densities LLIN usage was three-fold lower than that observed in the rainy season when mosquito densities were higher [8,9]. A net monitoring device could help verify survey data, and provide quantifiable net use data for periods between surveys.

Here we report a preliminary investigation into the potential use of an electronic motion detector/data logger attached to the LLIN to provide quantifiable data for monitoring LLIN usage.

## Methods

### Study area

The main movements of the LLIN linked to appropriate use for malaria control were characterised under laboratory conditions in Liverpool, United Kingdom in April 2009. Motion detectors were attached to the LLINs and the output data related to their movement on three axes (x, y, z) associated with specific movements. This was followed by preliminary field studies in two villages located in Toumodi (6°55N, -5°03W) and Yamoussoukro (6°582, -5°28W), in central Côte d’Ivoire, in May-June 2009. The field sites have high mosquito densities throughout the year. According to a 2009 census, both sites had approximately 70 households.

A recent study implemented in these villages [6] showed that large-scale distribution of LLINs, accompanied by training and sensitisation activities, significantly reduced clinical malaria rates over an entire 18-month period.

### Detector parameters

Unilever have developed a small electronic motion detector (logger) for use in large scale trials to assess usage patterns of soap, toothbrushes and other household consumer products. The nature of the consumer products that these motion detectors have been designed to be embedded within, require the detectors to be very small robust tamper-proof devices designed to withstand emersion in water. Unilever has made these proprietary loggers, and the associated software for data analysis, available to the Liverpool School of Tropical Medicine (LSTM) for the purpose of this project and any public health programmes. Depending on the periodicity of data collection and the expected frequency of the event to be measured, the battery life of the loggers varies from a month to several days. Data then needs to be downloaded and the loggers batteries recharged. This can be undertaken in the field. To recognise specific movements of the products the loggers are entrained through reproducing expected product movements and marking them in the software. Four volunteers carried out initial entrainment of the loggers, to measure movements associated with net use. LLIN use was simulated over a one month period and the data recorded by the data loggers used to characterise movements recorded when the nets were tied, untied and slept under or untied but not used.

Below, we described succinctly the configuration and use of the logger (as shown in Figures S1 and S2, further details are given in the Additional file 1).

To initialise the logger:

(i). A new battery is inserted into the logger (A) which is then connected to the serial port of the PC (D). The green LED (C) illuminates and the LogView application software is opened. The "**Get System Information**" button is pressed to display the current logger status and old data is removed using the "**Erase Data**" function. The logger settings are adjusted in the software and the settings transferred to the logger using the "Initialise Logger Function". The logger is disconnected from the PC and once the green LED (C) is extinguished, the logger is ready for use.

After incorporation onto the net for one week, the data was downloaded from the logger using the following procedure:

The logger is connected to the serial port of the PC and the green LED (C) should illuminate. Data is downloaded into the software using "**Get System Information**" function to display the current logger status then using the "**Save Data**" function to download the data. After saving the data file to the PC the "**Erase Data**" function is used to erase the logger’s memory so that it can be immediately reused.

### Collection of preliminary data with motion detectors

Data was collected from the detectors fixed to four nets used by volunteers selected in a village of Toumodi, central Cote d’Ivoire. Data was collected related to the movement against the x, y and z axes of the logger over a one month period. The volunteers using the nets were asked to fill in a questionnaire to indicate each day, the time at which the nets were tied when waking up in the morning, and when nets were untied before sleeping at night. They were also asked to indicate days when the nets were not used. The data recorded by the loggers was then correlated to the activities reported in the questionnaire. During this period, data from the loggers was downloaded weekly.

### Entomological surveys related to treated net use

Two entomological surveys, each lasting 6 days, were carried out in May and June 2009 in the village of N’Dakonankro located in Yamoussoukro, central Cote d’Ivoire. Adult mosquitoes were collected by six standard CDC light traps installed inside households with and without LLINs (PermaNet® 2.0). The surveys were conducted between 6.00 pm and 06.00 am.

In June 2009, a pyrethrum knock-down spray collection was performed inside six randomly selected sleeping rooms in addition to those where CDC light traps were installed [10]. Collections were undertaken in fifteen sleeping rooms with a total sampling effort of 30 sleeping rooms assessed over the two surveys. After collection, mosquitoes were sorted, counted and morphologically identified using an identification key [11].

To assess whether there was any difference in LLIN or untreated net use, in both sites, 20 untreated nets were offered to household members in addition to the LLINs already distributed. Households that received untreated nets were offered LLINs at the end of the study. With the help of the community health workers (CHWs), households involved in mosquito collection were selected randomly. Initialised detectors were fixed on donated (PermaNet® 2.0) and untreated nets used by households involved in the entomological surveys . The number of mosquitoes collected per household was then assessed relative to the effective use of the nets. Loggers were initialised or activated by the research team when initially visiting the households which were provided with nets.

### Monitoring of the use of LLINs in relation to the motion detectors

A pre-tested and validated questionnaire was conducted in Toumodi, central Cote d’Ivoire, every week after offering PermaNet® 2.0 (LLIN) to each household member in order to check when and at what time householders slept under the LLIN, or when the LLIN was tied or untied. When the questionnaire was administered the data loggers fixed on the LLINs were retrieved, data downloaded, the loggers battery recharged and the logger re-installed on the net. Data collected by the loggers was then analysed to assess its usage, i.e. the length of time the net was tied above the sleeping area, meaning it was unused, untied and used, or untied and not slept under.

Results obtained with the motion detector from each household were compared to those obtained from the questionnaire. The questionnaire was given to each household member sleeping under an LLIN to check whether they slept under a LLIN the previous night, the previous week, and the previous 2–4 weeks. This task was undertaken every week for a 6 week period by the Community Health Workers.

### Ethical issues

Before field work commenced, ethical clearance was obtained from the Ministry of Public Health and Hygiene through the National Malaria Control Programme. The study purpose, potential benefits and risks, operational procedures of the research i.e. the rights of study participants and obligations of the investigators, was explained to the local communities, local nurses, community health workers and religious leaders. Community meetings were held in the selected villages of the study area before the implementation of the trial. Signed informed consent forms were obtained from all households involved in the study. Interactions with communities were undertaken regularly.

The selected households and volunteers involved in mosquito collection were informed and invited to participate in the study via an informed consent form. The signed informed consents and assents were witnessed and translated in local languages. Participation in the study was entirely voluntary and each individual was free to leave the study at any time.

Mosquito collectors were protected against malaria by appropriate chemoprophylaxis.

### Data analysis

The data recorded was double-entered and cross-checked using MS Excel 2007 (Microsoft Corp, Seattle, WA). Analysis and comparison of categorical data such as rates of sleeping under treated nets or LLINs were performed with *χ*^2^ test by using version 10.0 of the STATA software package (Stata Corporation, College Station, TX). Data from the loggers was associated with the movements related to nets being tied, untied and used or untied and unused. Variability between the data recorded and expected standard deviations were established from entrained values from loggers fixed on untreated nets used by four adult volunteers for a one month period. Poisson regression models were fitted to compare the mean and overall number of mosquitoes collected with light traps and by pyrethrum knock down collections in sleeping rooms when the treated (PermaNet® 2.0) or untreated nets were tied, untied or not used. A likelihood-ratio test (LRT) was employed to explore the statistical significance between the mean and overall number of mosquitoes caught with each collection method. Standard deviations were calculated with data recorded by loggers when nets (untreated nets or LLIN) were tied, untied and not used. A P-value of 0.05 or less was considered indicative of a statistically significant difference.

## Results

### Patterns of net use

Movements associated with tying the nets after use produced standard deviations values reflecting the fact that tying a net is a long motion (8 to 20 seconds) necessitating many movements. From volunteer data, the minimum and maximum standard deviation values reported when nets were tied for x, y and z axes were 20.82 vs. 24.23, 26.04 vs. 60.66 and 23.16 vs. 67.77 (Table 1), respectively. In contrast, untying a net prior to use is a short motion lasting 0.5 to 4 second with low standard deviations. The minimum and maximum values of the standard deviations reported on x, y and z axes were 5.75 vs. 14.44, 3.67 vs. 13.9 and 2.69 vs. 12.91 (Table 1), respectively. Finally, data recorded when nobody was sleeping under an untied net showed extremely low standard deviations confirming the absence of movement. The minimum and maximum of the standard deviations reported with data downloaded on x, y and z axis were 0.66 vs. 3.82, 0.56 vs. 4.28 and 0.42 vs. 3.47 (Table 1), respectively.

**Table 1:** Standard deviations of the values on the x, y and z axis when movements were recorded associated with a net being tied, untied or unused by four adult volunteers over a 4 week period in Toumodi, Central Cote d’Ivoire

		**Untreated net tied movement**			**Untreated net untied movement**			**Unused untreated net movement**		
**Adult volunteers**		**Axis**			**Axis**			**Axis**		
		**X**	**Y**	**Z**	**X**	**Y**	**Z**	**X**	**Y**	**Z**
**Volunteer 1**	Day 1	36.33	38.75	37.44	9.31	6.17	4.088	1.69	1.01	0.95
	Day 2	42.23	44.03	34.59	13.87	6.64	5.761	0.858	0.63	0.592
	Day 3	36.62	26.81	24.046	8.055	9.329	6.38	1.317	1.444	3.107
	Day 4	23.001	50.603	25.409	9.17	6.52	8.79	1.444	4.2798	1.096
	Day 5	26.44	39.997	28.825	12.00	5.08	5.05	3.18	2.776	2.788
**Volunteer 2**	Day 6	36.357	26.609	23.167	6.19	3.67	4.49	1.29	2.25	1.67
	Day 7	34.22	58.13	37.15	8.05	6.58	2.69	1.32	1.70	0.57
	Day 8	20.82	26.04	29.16	6.96	9.53	9.39	0.68	0.56	0.61
	Day 9	25.81	28.99	31.53	6.79	6.68	7.00	0.66	0.65	0.93
	Day 10	37.05	53.81	58.05	8.05	13.90	9.66	2.42	1.97	1.80
**Volunteer 3**	Day 11	40.01	41.15	67.66	10.21	8.55	5.54	2.40	0.92	1.47
	Day 12	26.04	37.08	58.45	11.01	6.31	11.26	3.31	3.00	2.49
	Day 13	35.37	41.38	43.92	6.82	11.79	5.46	1.20	0.78	1.83
	Day 14	40.13	47.87	57.49	10.42	8.02	11.01	3.82	2.1	1.66
	Day 15	30.99	42.2	56.25	14.1	6.167	12.91	0.66	0.77	0.42
**Volunteer 4**	Day 16	34.63	40.60	56.65	5.756	8.08	8.59	3.65	1.25	3.47
	Day 17	36.00	46.67	57.90	14.44	8.99	7.646	2.83	1.31	2.08
	Day 18	40.41	60.66	63.03	11.66	6.39	10.94	3.28	1.01	2.47
	Day 19	33.85	48.15	53.88	11.15	9.85	12.56	1.44	1.99	1.51
	Day 20	30.99	36.98	35.42	10.35	8.59	7.41	2.67	1.97	3.46
**Minimum**		20.82	26.04	23.167	5.756	3.67	2.69	0.66	0.56	0.42
**Maximum**		42.23	60.66	67.66	14.44	13.9	12.91	3.82	4.2798	3.47
**Mean**		33.36	41.82	44	9.71	7.84	7.83	2.006	1.62	1.748

The values recorded for the x, y and z axes given in Table 1, do not overlap for the movements of tying, untying and not using a net, allowing all three movements to be unambiguously detected. Thus, based on this data, it is possible to specify x, y and z values for the net use pattern with a high degree of (100%) specificity and (100%) sensitivity e.g. all x values greater than 19 indicates that the net is tied.

### Comparison of reported and logged LLIN use

In the Cote d’Ivoire study site, logger data was compared to questionnaire results (Table 2). One week after net distribution, net use recorded by questionnaires was overestimated by 13.6% compared with the logger data. This overestimate increased with time, the overestimates were 22.8%, and 38.7% when questioned about net use two and four weeks previously.

**Table 2:** Estimation of the proportions of net use within household members by using data loggers and questionnaires in four villages located in Toumodi, central Cote d’Ivoire

**Net usage indicators**	**Previous night**	**Previous week**	**2 weeks ago**	**3 weeks ago**	**4 weeks ago**
Proportion of people sleeping under LLIN estimated **by questionnaires** (%)	81.9 (36/44)	72.7 (32/44)	68.2 (30/44)	68.2 (30/44)	59.1 (26/44)
Proportion of people sleeping under LLIN estimated by **data loggers** (%)	81.9 (36/44)	59.1 (26/44)	45.4 (20/44)	36.4 (16/44)	20.4 (9/44)
**Overestimated rate of nets usage (%)**	0.0	13.6	22.8	31.8	38.7

### Mosquito biting activities in relation to net use

In order to check whether there is any difference in the number of *An. gambiae* collected in sleeping rooms where treated (PermaNet® 2.0) or untreated nets were used, mosquitoes were collected using CDC light traps or pyrethrum knock-down collections. Data recorded with detectors enabled the research team to determine when nets were tied, untied or not used (Table 3).

**Table 3:** Number of mosquitoes collected with CDC light traps and pyrethrum knock down in relation to net movement when used or unused in Yamoussoukro, central Cote d’Ivoire

**Mosquito collection method**		**LLIN**			**Untreated net**		
		**Tied**	**Untied**	***P-value***	**Tied**	**Untied**	***P-value***
**Light trap**		72	18	<0.001	123	61	<0.001
**Pyrethrum knock down collection**	Number of blood fed	97	31	<0.001	114	56	<0.001
	Number of Unfed & gravid	128	56	<0.001	278	136	<0.001

The mean number of *An. gambiae s.s* mosquitoes collected in CDC light traps in households where an LLIN was untied was significantly lower than the mean number of mosquitoes collected in households where the LLIN was tied (LRT = 273.70; *P* < 0.001). Similarly, the mean number of *An. gambiae s.s* collected in CDC light traps in households where the untreated net was untied was significantly lower than the number of mosquitoes collected where the untreated net was tied (LRT = 68.91; *P* < 0.001).

As expected, untied nets give better protection against blood feeding than tied nets, as significantly more blood fed *An. gambiae s.s* were collected in sleeping rooms with tied nets compared to the number collected in sleeping rooms where the LLIN was untied (LRT = 119.85; *P* < 0.001). A similar trend was seen with untreated nets (LRT = 217.03; *P* < 0.001). There was also a significant difference between the numbers of *An. gambiae s.s.* collected in sleeping rooms where the treated net was untied compared to the sleeping rooms without nets (LRT = 134.85; *P* < 0.001).

## Discussion

This study provides initial validation of the potential for the use of motion detectors to accurately monitor the usage of LLIN by household members. This is an innovative technique which allows output data from the detectors to be associated with specific movements related to LLIN use e.g. tying and untying the nets, compared to nets remaining unused. The detector can be used to generate quantitive measures of net usage and could, with further development, offer validation of current questionnaires and surveys.

Output data from this study indicated an overestimate of net usage by questionnaire with estimates increasing as the time between the questionnaire and the act of sleeping under the net increases. Reported net use 24 h before the survey recorded by detectors and questionnaires were similar. LLIN usage rates reported by questionnaire were sometimes overestimated if householders were questioned more than one week after use. In Senegal and Ethiopia, recent studies demonstrated that 34.7% [12] and 36.1% [13] of surveyed individuals had slept under treated nets the previous night, which was lower than desired, despite high net ownership. This is the most commonly used Roll Back Malaria indicator used by all malaria control programs in endemic countries to assess effective usage of LLINs. This indicator seems to be working well. Low LLIN use reported in numerous studies [13,12,14] may be influenced by housing characteristics, mosquito abundance and human behaviour. Houses which have closed eaves and/or ceilings have fewer mosquitoes than those with open eaves [15]. The external environment may also influence mosquito densities [16].

Use of loggers would allow seasonal variations in net usage to be monitored routinely, compared to one off questionaires asking about net usage over time. A recent survey conducted, in Kenya, Madagascar, Niger, Sierra Leone and Togo showed the limitation of net usage surveys conducted weeks or months after LLIN distribution. The timing of surveys three to nine months after the nationwide campaigns could introduce bias, if respondents had difficulty remembering events from the campaign, misclassified the net type, or over-reported net use on the basis of social desirability [17]. Detectors could help to address such issues.

## Conclusion

Accurate reporting of LLIN use will be needed to support malaria elimination efforts, especially in endemic countries where net usage is influenced by several external factors. There is a need to find appropriate tools that will help to record exactly when nets are properly used. The detectors used in this study could be used to assess actual LLIN use. More emphasis needs to be placed on the fact that malaria transmission occurs all year round thus there is a need for regular protection. The use of data loggers to efficiently quantify the usage of LLINs within households provides a non-invasive method of monitoring net use. Additionally, data loggers can be used to verify survey data, reducing the need for routine household visits. With further development, they should be considered as complementary tools to the existing methodology used in social science. The main problem of this generation of detector is the relatively short life of the battery used. But a new generation of detectors has recently been developed with a longer lasting battery and greater data storage capacity, which could extend the feasible use of this new monitoring tool.

## Abbreviations

CDC: Centre for diseases control; LLIN: Long Lasting insecticide-treated Net; CHW: Community health worker.

## Competing interests

The software used to analyse data collected during the implementation of the project was patented by Unilever. Unilever has agreed that LSTM has the right to use the software for an indefinite period to evaluate the use of vector control products from any source. The authors declare no competing interests.

## Authors’ contributions

BGK designed experiments, coordinated field activities, collected and analyzed data, wrote and revised the paper; DM supported the study design, reviewed the manuscript; JH designed the study, participated in the coordination of field activities and revised the paper. All authors have read and agreed with the content of the submitted manuscript.

## Supplementary Material

Additional file 1: Figure S1 Presentation of the main parts of the data logger used in this preliminary study. **Figure S2.** Below depicts a screenshot of the LogView application. A summary of the key features of the LogView software is provided below. Click here for file
