# Supplementary material for: The use of motion detectors to estimate net usage by householders, in relation to mosquito density in central Cote d’Ivoire: preliminary results
Source: Parasit Vectors. 2014 Mar 6;7:96. doi: 10.1186/1756-3305-7-96 (PMC4015446; doi:10.1186/1756-3305-7-96)
Supplement: Additional file 1: Figure S1 — Presentation of the main parts of the data logger used in this preliminary study. Figure S2. Below depicts a screenshot of the LogView application. A summary of the key features of the LogView software is provided below. [file 1756-3305-7-96-S1.doc]

Additional file 1

This appendix provides guidance on the configuration and use of the logger (shown in Figure 1 below). The device records 3-axis acceleration data and is intended for use in consumer trials such as nets trial.

**1. Characteristics, Description and use of the data logger**

*Usage Profile*

• The user can optionally specify the following logger parameters:

• Sampling frequency (integer frequencies between 1 and 100Hz).

• Trigger level (integer values between 1 and 1000mg). The trigger level specifies the absolute level of acceleration (on any axis) necessary to trigger a recording. (A level of 100mg has been used successfully in the past.)

• Record length (integer values between 1 and 120 seconds). The record length specifies for how long the logger will record data after being triggered.

• Daily record limit. The daily record limit specifies the maximum permissible recordings per calendar day or months. Once the daily record limit has been reached, the logger will ignore all subsequent events until the next calendar day or months. To specify no daily record limit, set the value to zero.

• Current parameter values can be obtained at any time by pressing the “**Get System**

**Information**” button.

*Configuration of a usage Profile*

• To recall an existing profile, simply select the desired profile from the drop-down list. The parameter values will change to reflect the new profile. Note however that the parameters selected are not written to the logger until the “**Initialise Logger**” button is next pressed.

• To delete an existing profile, select the profile as above, and press the “**Delete Profile**” button.

• To create a new profile, first enter the desired parameter values. Type in a name for the new profile (or edit an existing profile name) in the drop-down profile box. (Profile names can be up to 15 characters long). Press the “**Save Profile**” button.

• Profile information is stored in a separate text file called “**LogProfile.txt**” which must reside in the same directory as the LogView executable. Profiles can be transferred between different PCs by copying the LogProfile file.

*System Time*

• Press the “**Get System Information**” button to display the current time and date according to the logger’s on-board clock.

• To set the time and date, either

• Tick the “**Use PC clock to set time**” check-box. The logger’s on board clock will be synchronised with the host PC; or

• Untick the “**Use PC clock to set time**” check-box and use the two arrow controls to select an arbitrary date/time.

Note that in both instances the logger’s clock is not actually updated until the “**Initialise Logger**” button is next pressed.

*Memory Status*

• The memory status display provides a visual representation of logger memory usage. Press the “**Get System Information**” button to update the memory status display at any time.

• Press the “**Save Data …**” button to download recorded data to the PC.

The download time varies according to how much data is stored in the logger. It may take up to 15 minutes to complete download if the memory is full.

• Once the data has been downloaded, the user is prompted to enter a filename for the data. By default, LogView stores the saved data in comma-separated variable format (.csv) for compatibility with a wide variety of data analysis programs (including Excel).

• If the chosen file already exists, the user will be asked if he/she wishes to overwrite the existing file. If the chosen file cannot be opened for any reason, or is already open in another application (e.g. Excel), LogView will display an “Invalid File” warning.

• Downloading data does NOT clear it from the logger’s memory.

• Press the “**Erase Data**” button to erase the logger’s on-board memory. A dialog box will be displayed asking the user to confirm the erase. The memory status display is automatically updated.

*Get System Information*

• Press the “**Get System Information**” button at any time to display the current logger status as described above.

***Initialise the Logger***

**• Press the “Initialise Logger” button to transfer the displayed settings to the PC.**

• If the logger is not blank (i.e. there is still data in the logger’s memory), the user will be asked to confirm the operation.

• The logger makes a special entry in its memory each time the “**Initialise Logger**” button is pressed, recording the time and date of initialisation. This information is included in the output data file.

**2. Usage Notes**

• The logger is designed for use with a Renata CR1225 battery. Use of a different battery will impair performance and may be hazardous.

• If connected to a PC, the green LED (C) will illuminate when the logger is ready to communicate.

• Connecting the communications cable will immediately terminate any recording in progress.

**3. Logger Initialisation Procedure**

The main steps to initialise the data logger are:

1. Insert a new battery into the logger (A).

2. Connect the data logger to the serial port of the PC using the supplied cable (D).

If the PC has more than one serial port, ensure the logger is connected to COM1. Ensure that the supplied external power supply is connected to the cable and switched on. Do not leave the logger connected to the PC without an external supply for any longer than is necessary.

3. Wait for the green LED (C) to illuminate.

4. Start the LogView application of the PC (if not already open).

5. Press the “**Get System Information**” button to display the current logger status.

6. Press the “**Erase Data**” button to remove any remanent data.

7. Adjust the logger settings as desired.

8. Press the “**Initialise Logger**” button to transfer the settings to the logger.

9. Disconnect the communications cable from the data logger.

10. Wait for the green LED (C) to extinguish.

11. The logger is now ready for use.

**Figure S1:** Presentation of the main parts of the data logger used in this preliminary study

**4. Download Procedure of data recorded by the Logger**

1. Connect the data logger to the serial port of the PC using the supplied cable (D). If the PC has more than one serial port, ensure the logger is connected to COM1.

A battery is not required to download data but it is recommended that a battery is left in, so that the logger retains time and date and memory status information.

2. Wait for the green LED (C) to illuminate.

3. Start the LogView application of the PC (if not already open).

4. Press the “**Get System Information**” button to display the current logger status.

5. Press the “**Save Data**” button to download the data.

6. Enter a filename for the data when prompted.

7. Once the download is complete, press the “**Erase Data**” button to erase the logger’s

memory (if desired).

8. If the logger is not intended for immediate re-use, you may wish to remove the battery from logger (if present) to prevent unwanted recordings.

9. Disconnect the communications cable from the data logger.

The data logger is configured for use using “**LogView**” (software presented below) – a PC application which communicates with the logger via the PC’s serial port. The LogView software is also used to download logged data.

LogView is a self-contained Windows executable. As such, it requires no installation. To start LogView, simply double-click on the LogView icon.

Figure S2 below depicts a screenshot of the LogView application. A summary of the key features of the LogView software is provided below.

**Figure 2:** View of the LogView software used to download data recorded by the logger

**5. Troubleshooting**

• If, after sending a command to the logger, LogView receives an incorrect or no response from the data logger within a set period of time, a “**Communications Error**” message will be displayed.

• If a communication error occurs, try pressing the “**Get System Information**” button. If the communications link is working correctly, the clock display should immediately change to show the time according to the logger’s on-board clock.

• If the communications errors persist, shut down LogView and then restart it (leaving the logger connected).

• If the communications errors still persist, disconnect the logger from the PC and remove the battery (if fitted). After 30 seconds, re-connect the logger to the PC (ensuring that the external power supply is connected to the cable and switched on). Wait for the green LED to illuminate and then restart LogView.
